# Yoga intervention for colorectal cancer survivors: a qualitative study exploring participants’ expectations and experiences

**DOI:** 10.1080/07853890.2024.2397571

**Published:** 2024-08-30

**Authors:** Mirela-Ioana Bilc, Nina Pollmann, Clemens Eisenmann, Analena Buchholz, Bijay Pokhrel, Romy Lauche, Holger Cramer

**Affiliations:** a Institute of General Practice and Interprofessional Care, University Hospital Tübingen, Tübingen, Germany; b Robert Bosch Center for Integrative Medicine and Health, Bosch Health Campus, Stuttgart, Germany; c Department of Sociology, University of Konstanz, Konstanz, Germany; d Medical Care Center of Recura Clinics, Beelitz-Heilstätten, Germany; e National Centre for Naturopathic Medicine, Southern Cross University, Lismore, NSW, Australia

**Keywords:** Colorectal cancer, yoga, qualitative study

## Abstract

**Introduction:**

Colorectal cancer (CRC) survivors often struggle with side effects following treatment such as reduced quality of life, fatigue and psychological distress and need therefore efficient comprehensive interventions. The aim of this qualitative study was to explore CRC survivors’ expectations before the yoga intervention as well as their unique experiences beyond those reported with standard questionnaires.

**Methods:**

Interpretative phenomenological approach was used in this qualitative study. Semi-structured interviews were conducted before and after a 10-week yoga program (90 min once a week, Hatha Yoga) with CRC survivors enrolled in a randomized controlled trial. Thematic analysis was used to uncover themes present in participants’ accounts.

**Results:**

Nine patients participated in the interviews, mean interview duration was 27.49 min (SD = 7.71) before and 38.41 min (SD = 15.93) after the intervention. Our analysis identified following themes: (1) representations and expectations from the yoga intervention; (2) course structure and implementation; (3) perceptions and effects of the intervention; (4) differences between the study yoga intervention and other physical activities. The superordinate theme regarding effects of intervention included aspects of intervention at multiple levels such as emotional, physical, behavioral and spiritual.

**Conclusions:**

This qualitative study provides valuable insight regarding CRC survivors’ expectations and experiences following a 10-week yoga intervention. While expectations varied from skepticism to specific symptom improvement, the majority of participants had a positive, open attitude towards yoga. Consistent with participants’ experiences, yoga may represent a promising intervention for CRC survivors if the groups’ specific concerns are taken into account.

## Introduction

Colorectal cancer (CRC) is the third most common cancer diagnosed worldwide and the second-leading cause of cancer deaths, with both incidence and mortality rates higher in males than females [1]. Based on rates from 2020, estimates of CRC burden predict that by 2040 the number of new cases will increase to 3.2 million and 1.6 million deaths [2]. The American Cancer Society [3] and the US National Cancer Institute [4] use the term cancer survivor from the time of diagnosis onward, irrespective of treatment stage. We adopted this broad-sense definition for the rest of this paper. Advancements in cancer prevention, diagnosis and treatment are associated with increased survival rates, however survivors often struggle with side-effects during treatment as well as after treatment completion. These include among others deterioration of health-related fitness, reduced quality of life, fatigue and psychological distress [5–9]. A recent meta-synthesis of 15 qualitative studies on CRC survivors’ experiences of long-term impacts on health-related quality of life highlighted the major role played by persistent gastro-intestinal and functional impairments alongside other psychological, social and work-related effects [10]. Patients also reported unmet needs and uncertainty in managing these long-term symptoms. This underlines the necessity of finding comprehensive interventions, which address the wide array of difficulties that cancer survivors need to face. Mind-body therapies, such as yoga are promising candidates.

Yoga is an ancient traditional spiritual practice rooted in Indian philosophy, whose modern practice commonly includes physical postures (asanas), breathing techniques (pranayama) and meditation (dhyana) [11]. Yoga has become a popular means to promote physical and mental well-being [12] and was shown to have positive effects on cancer survivors’ quality of life and psychological health [13–16], fatigue [17,18] as well as insomnia [19], but most of the existing evidence relies on data from breast cancer survivors. These results are nonetheless encouraging given that long-term symptoms such as prolonged fatigue, cognitive limitations, depression, anxiety, sleep problems, pain and sexual function are common among both breast and colorectal cancer survivors [20]. However, little is known about the efficiency of yoga for CRC survivors, especially for specific symptoms such as bowel problems that strongly influence quality of life [21]. There are also differences in patient profiles with a higher incidence of CRC among males and a peak incidence in individuals aged 60–74 years old [22], while 99% of breast cancer patients are female [23] and 70% of all new cases are aged 50 and above [24] Considering that female sex and younger age are associated with increased likelihood of practicing yoga [12] it is worth considering how the CRC patient profile could impact the implementation and effectiveness of yoga interventions. To address this gap, we conducted a randomized controlled trial (RCT), with the aim of investigating the effects of a 10-week traditional yoga intervention on health-related quality of life in CRC survivors. Using disease-specific questionnaire, we found no effect of yoga on health-related quality of life in this study [25]. While RCTs are considered the gold standard to assess the effectiveness of an intervention, there are multiple ways in which they can benefit from the use of additional qualitative measures [26]. While qualitative studies have been extensively used to explore experiences of breast cancer survivors with yoga [27], less is known about CRC survivors. This also applies to other interventions, with a meta-synthesis of qualitative studies on physical activity and quality of life in cancer reporting 19 studies on breast cancer survivors and only 2 on CRC survivors [28]. As regards other mind-body practices, Wan et al. [29] conducted a qualitative exploration of mindfulness and qigong practices for CRC survivors indicating overall positive effects, as well as specific effects. Participants practicing qigong reported physical improvements, while for those practicing mindfulness, improvements were rather in terms of spiritual growth and intrapersonal connectedness.

Mackenzie et al. [30] described the experiences of different types of cancer survivors, CRC included, and their support persons enrolled in a community-based yoga program using a focus-group approach. The authors found that cancer survivors and their support persons experienced beneficial effects of the yoga intervention and emphasized the role of increased awareness of mind-body connection and improvements in breath regulation as possible mechanisms of action. While informative, there are differences between community-based programs and intervention programs embedded in clinical trials, which could potentially influence participants’ experiences.

Taking all of the above into account, our goal in the present qualitative study was to gain insight into CRC survivors’ expectations before the yoga intervention as well as their unique experiences during the intervention, which might otherwise be difficult to capture by standard questionnaire measures.

## Methods

### Study design

A qualitative research methodology guided by an interpretative phenomenological analysis (IPA) framework was employed in this study. IPA is theoretically grounded in phenomenology and allows researchers to gain insights into the unique perspective of participants [31].

Data reported in this study were collected as part of a larger open-label, randomized controlled bicenter clinical trial whose quantitative results have been previously reported [25]. Semi-structured interviews were conducted with participants from the wait-list group, who were offered the yoga intervention following end of data collection. The yoga intervention consisted of weekly, 90-minutes Hatha Yoga over a period of 10 weeks. The study was approved by the Ethics Committee of the University of Duisburg-Essen (approval number 12-4957-BO) and the Charité University Medical Center and registered at clinicaltrias.gov (NCT01669109) before patient recruitment. This study adheres to the Declaration of Helsinki regarding research involving human subjects. Written informed consent was obtained prior to study participation.

### Participants

Participants for the RCT were recruited by the study physician from the Department of Surgery and Center for Minimal Invasive Surgery, Evang. Kliniken Essen-Mitte, Essen, Germany and the Tempelhof Colon Center, St. Jospeh’s Hospital, Berlin, Germany. Patients, male and female, were included if they were at least 18 years old and have had surgical treatment for histologically confirmed non-metastatic CRC (stages I–III) in the previous 48 months. Exclusion criteria referred to physical disability precluding even light yoga practice, regular yoga practice within the prior 12 months, further active oncological disease, diagnosed and pharmacologically treated psychiatric disorder except cancer-related depression or adjustment disorder, pregnancy and breastfeeding.

### Data collection

All RCT participants from the wait-list group who underwent the yoga intervention at the study center Essen were invited to participate to semi-structured interviews before and after the intervention. The underlying consideration was to interview CRC survivors whose yoga participation was associated as little as possible with the rest of the research process. At the time of the interview, all quantitative data collection had been completed. The first interview focused mainly on exploring CRC survivors’ representations and expectations regarding yoga therapy, while the second interview addressed impressions about the yoga intervention, changes in body perception and health management as well as emotional, social and spiritual factors. A semi-structured interview guideline was prepared in advance and used during interviews (Supplementary Appendix A). Participants were also given the opportunity to address aspects that they felt were particularly important and which may not have been asked directly in the interview guide.

The interviews were conducted by two PhD-level psychologists (R.L. and H.C.) and one MSc-level sociologist (C.E.), who had received formal and informal training in qualitative methods and conducted qualitative studies before. The interviews were held in a private room at the Department of Integrative and Internal Medicine, Evang. Kliniken Essen-Mitte, Essen, Germany before and after the yoga intervention. The interviews were pseudoanonymously audio recorded and then transcribed verbatim. Overall 527 min of data were collected, with a mean interview duration of 27.49 min (SD = 7.71) before the intervention and 38.41 min (SD = 15.93) after the intervention. None of the interviewers had any contact with the study participants before the first interview. No field notes were taken. No participant-checking was adopted.

### Data analysis

The interviews were analyzed by the three interviewers and two medical students (N.P. and A.B.) who had no contact with the participants at any time. Thematic analysis, a method that aligns with the underlying assumptions of IPA was employed for data analysis and theme identification. The transcribed text files were uploaded into the MAXQDA program, which was used to code, analyze, and summarize the data. Before the first text analysis, two independent coders discussed and decided upon a list of pre-set codes based on the interview guidelines and topics of interest. This served as orientation for the first text analysis, which encouraged inductive coding and thus further development of codes. A second text analysis took place with renewed assessment by the same two independent coders of the individual text passages and assignment of the adjusted codes. The independent codes were then merged into a single version after discussion and adjustment of discrepant classifications and a final crosscheck. Subsequently, these codes were combined into superordinate themes, primarily by means of inductive category formation. These individual steps took place in regular exchange and discourse with members of the working group.

### Trustworthiness

A series of aspects were taken into account to help strengthen the credibility, transferability and relevance of this study. While participant-checking was not adopted, peer-debriefing was used at all steps of data analysis. These reflexive discussions allowed researchers to take into account different perspectives and interpretations of data. In addition, participants’ quotes were used to illustrate identified themes, at the same time allowing the readers to make their own interpretation. Furthermore, both demographical and clinical information about participants was provided for each quote thus further helping readers to evaluate the transferability of these findings.

## Results

### Participants

Nine participants attended the semi-structured interviews, 7 of which both before and after the yoga intervention, 1 only before and 1 only after the intervention. Participants were aged 63–74 years old (MD = 69.66, SD = 4.02), with a relatively equal sex distribution (4/9 females). Five participants had stage I or II cancer, while the remaining four had stage III cancer. As regards cancer type, 7 of them had rectal cancer and 2 had colon cancer. Moreover, regarding cancer treatment, 5 reported prior chemo- and radiotherapy, 1 reported only prior chemotherapy while 3 had neither. None of the participants was currently undergoing chemo- or radiotherapy. Time since surgery ranged from 9 to 46 months (MD = 26.44, SD = 13.21) and 3/9 participants underwent colostomy. Participants’ characteristics are presented in Table 1.

**Table 1.** Characteristics of study participants.

ID	Sex	Age (years)	Cancer type	Cancer stage	Prior chemotherapy	Prior radiotherapy
1	Male	64	Rectal	I	Yes	Yes
2	Male	72	Colon	I	No	No
3	Female	63	Rectal	III	Yes	Yes
4	Male	69	Rectal	II	Yes	Yes
5	Female	67	Rectal	III	No	No
6	Male	74	Colon	III	Yes	No
7	Female	74	Rectal	III	Yes	Yes
8	Female	74	Rectal	I	Yes	Yes
9	Male	70	Rectal	II	No	No

### Themes

#### Representations and expectations from the yoga intervention

Very few participants had previous explicit experiences with yoga. Those who had contact with yoga elements during rehabilitation therapy or independently performed exercises using books reported positive experiences, often mentioning an intensified body perception. Previous experiences with relaxation practices and body exercises in the context of Pilates, Qi Gong or Tai Chi were rather unfavorable.

Representations of yoga varied between individuals. While the majority associated yoga with gymnastic exercises on a mat in combination with breathing exercises, there were also individual views that yoga was primarily associated with spirituality and concentration and that body awareness exercises were a large part of it. The partial lack of yoga knowledge sometimes resulted in reserved expectations towards the intervention. The basic attitude was mostly open and positive. Some hoped for symptom improvement such as digestive problems or dealing with pain:

I’m having a bit of trouble with my bowels at the moment […] relaxation is good to be able to process pain better or things like that, and also the exercises that possibly get the intestines moving a bit, that they do me good. [ID 1, male, 64 years].

Others hoped for help with adjusting to everyday life, a general improvement in well-being through inner peace and relaxation or improving their body perception. Skepticism about the occurrence of a positive effect, was also present, especially in patients who complained of only minor residual symptoms.

So I had few expectations of my illness, because I’ve had zero problems so far and no problems now either [ID 8, female, 74 years]

Nevertheless, these participants wanted to experience it on their own and then form their opinion. The majority looked forward to the course with anticipation and curiosity. Regarding possible mechanisms of action, the effect of yoga was described as mind-body congruence that could have an effect on the intestinal activity and help in dealing with the pain:

I always think that you feel good when your body is in order and your soul is in harmony. If it’s not, just one of the two, it doesn’t work. So it has to be in harmony, for body and also for the soul. [ID 5, female, 67 years]

Other expectations regarded self-discovery, achieving a more balanced and deeper breathing and gaining more elasticity of the ligaments by stretching them.

For the most part, CRC survivors had no reservations about taking part in the course. Individual concerns related to the stoma becoming loose, the possibility of unpleasant situations due to incontinence issues, fears of exertion or difficulties due to limited mobility.

Reasons for study participation, included the fact that yoga was something that participants felt they could do despite certain physical limitations, that they knew that exercise had positive effects and enjoyed movement, as well as curiosity about possible benefits. Another point mentioned was that they wanted to draw attention to CRC which they felt was underrepresented compared to breast cancer and therefore to help future CRC survivors.

#### Course structure and implementation

Some participants reported having difficulties and needing adjustment for some of the yoga postures. It was often mentioned that the yoga teacher responded promptly and adapted the execution to each participants’ abilities. This was valued several times and contributed significantly to participants feeling they were well taken care of. Due to the stoma, some movements were not feasible and some participants have reported having muscular discomfort during the first days of the course, which however, quickly subsided. The physical exertion of yoga was surprising for some, as was the fact that they were confronted with their own limitations, some of which they were not aware of. The course emphasized therefore the need for movement and training. The difficulties in performing the exercises due to their physical condition were so serious for some patients that they decided against continuing yoga after the course. One patient described this as follows:

It was practically everything I couldn’t do. I couldn’t even lie on the hard mat. Whether I was in rehab or wherever, I was given a pressure sore cushion straight away. [ID 7, female, 74 years]

Regarding the course structure, participants reported expecting more static exercises and therefore the dynamic in many exercises was a positive surprise. However, critic was expressed for not integrating enough exercises with an explicit reference to the bowel. During the breathing exercises and meditation, several participants mentioned thought wandering and difficulties in letting go completely. The preference for specific exercises varied greatly, with some focusing more on relaxation and meditation, while others found the physical exercises especially important. The sun salutation as a combination of different exercises was perceived as a focal point and could be integrated well into everyday life.

So, the sun salutation was the highlight, I’d say, because everything was there in combination. [ID 8, female, 74 years]

The majority of participants evaluated the group format of intervention positively. Seeing that everyone had difficulties from time to time has prevented them from losing courage and motivated them to continue and make progress. Some emphasized the importance of the fact that all participants had the same illness. This created an atmosphere of consideration, there was no pressure to perform and in contrast to a regular course, there was no need to worry about being overwhelmed:

I know that if I went to a normal yoga class, I would be out of place. Here that [the illness] was taken into consideration. [ID 2, male, 64 years].

The yoga course had given room to talk about partly unspoken problems related to the disease and therapy. Through the exchange of experiences, one could get helpful tips on how to deal with them.

#### Perceptions and effects of the intervention

##### Emotional level

The majority of participants noticed that yoga was good for them and that they felt better afterwards. Their well-being increased and they perceived that after yoga everything in the body was mobilized. Meditation in particular, triggered unexpected pleasant feelings. A special sensation was mentioned during the breathing exercises as one consciously perceived the path of one’s breath in the body. This made some participants anticipate the positive effect of yoga and therefore they were looking forward to the course:

Now that [yoga course] comes back, when I’m all relaxed, when I’m all relaxed, that’s kind of like you’ve been given new life [ID 2, male, 72 years].

##### Physical level

Yoga had an influence on physical complaints as well as on one’s own body perception.

With regard to cancer-specific complaints, some participants described positive effects regarding digestion, with more regular digestive times, which facilitated organizing their daily routine, less extreme changes between constipation and diarrhea and less pain:

These cramps are during yoga and after that, uh, they decreased [ID 1, male, 64 years].

Others report not noticing any change, which they partly explained due to the time discrepancy between the disease and the start of the course or having a limited number of complaints anyway.

Participants also noticed improvements in other aspects such as reduction of muscle and joint pain, the release of muscle tension and the improvement of balance and overall physical condition:

I don’t have pain in my back anymore! Do you know what that’s like? It’s like I’ve had a second life built into me. Now that may sound a little bit too much, but it’s the truth. It’s the truth. [ID 2, male, 72 years].

And this yoga, I mean, it helps that my whole ‘framework’ becomes more stable and that I, uh, have a different body posture [ID 8, female, 74 years].

One participant, on the other hand, had negative experiences with yoga as the exercises had triggered severe abdominal cramps and pain. She described that, despite instructions to maintain her limits, she had over exhausted herself and finally decided to discontinue the course prematurely, but to remain available for data collection.

Some participants experienced a fundamental change in body perception, such as one participant who described:

I have rediscovered my body. It’s simply fantastic. [ID 2, male, 72 years]

Others however did not experience a major influence of yoga or cancer on their body perception.

Practicing for an hour does not do as much for me as going into the garden for an hour. […] You also have to bend down etc., but then I notice my muscles and of course the lazy spots that I have in the meantime, I notice them more than with yoga, where they say you shouldn’t overstretch or do it at all costs. [ID 4, male, 69 years]

##### Behavioral level and everyday life

Some participants mentioned having reconsidered their attitude to life as part of the yoga intervention. For others this had already happened after having cancer and the intervention was a confirmation. They no longer took the functioning of their own bodies for granted and have learned to be more mindful of themselves and their bodies:

Overall a little more mindful of one’s body, I guess I can say that. It’s not always easy, but that’s the way it is […] and that you’ve noticed that you also have to do something for your body. [ID 9, male, 70 years]

One participant described that through yoga she was able to deal more calmly and patiently with others and with everyday problems. In addition, others mentioned that yoga motivated them to change their eating habits.

For the duration of this study, the majority of participants stated that they had also practiced yoga regularly at home, sometimes even joined by spouses or grandchildren. Most of them found their experience with yoga to be very positive and therefore decided to continue even after completing the intervention, but there were also others who did not, reporting difficulties and fear of practicing yoga without supervision. As regards the integration of yoga in everyday life, examples include integrating postures in order to loosen up the body after certain tasks such as gardening, or doing the breathing exercises when having difficulties to fall asleep. Some also integrated yoga exercises firmly into their daily routine, such as while brushing their teeth.

##### Spiritual level

Spirituality was of secondary importance to the majority of participants, with a few exceptions. One participant reported having originally found access to yoga through spirituality, and another reported hoping to experience dimensions beyond the purely physical in the context of yoga classes. Others just had a basic open attitude toward the spiritual dimension of yoga. The connection between faith and yoga was particularly evident in the context of meditation:

But of course it can have something to do with faith in the sense that yoga is a way of finding the right meditation[…]Yes, I mean, becoming calm and concentrating on something higher and so on, yoga is certainly useful for that. [ID 9, male, 70 years].

#### Differences between the study yoga intervention and other physical activities

In contrast to many other physical activities, yoga did not arouse false ambitions and participants were always reminded to maintain their own limits. They reported that it became easier as they progressed and the effects lasted for a while after completion of the course. Some expressed the opinion that offering such a course directly after therapy would be more useful. Yoga was considered an ideal way of gently regaining strength after the strenuous therapy.

So, if that would have been there immediately, I think that would have contributed certainly even more to the recovery[…] That one regains ones strength after the chemotherapy [ID 3, female, 63 years]

## Discussions

In light of the limited amount of data on yoga interventions for CRC survivors, our qualitative analysis provides valuable insights into CRC survivors’ representations and expectations, as well as reported effects following a 10-week yoga intervention.

Our findings reflect some important aspects regarding conceptualizing and implementing yoga interventions. First, the group format was valued by most participants, as they felt understood and comfortable being surrounded by others who shared the same disease and benefited from the information exchange. Comparable experiences were reported for cancer patients [32,33] as well as patients with other medical conditions [34]. Second, this also signals potential barriers to transferring the intervention outside of the clinical setting which echoes previous findings on CRC survivors regarding physical activity in general [35–37]. Given specific constrains, CRC survivors might feel uncomfortable to take part into regular yoga classes and therefore need individualized interventions. This is further supported by reports of needing to adjust exercises to specific limitations. The instructor’s willingness to adjust was a deciding factor in them feeling safe and motivated to continue the intervention. Third, our results show that some components of the intervention such as specific postures and breathing exercises were more likely than meditation to be integrated in everyday life. On one hand this is encouraging and shows the practical relevance of yoga interventions, however it also signals that meditation might require extra scaffolding to ensure its successful home use [38]. Future studies or yoga courses could integrate this knowledge by allocating more time to meditation in class, as well as providing participants with additional materials to encourage home-use.

As regards the effects of the intervention, our results show that overall the yoga intervention was evaluated positively, with specific outcomes varying across individuals from physical to emotional and behavioral changes. This is in line with recent reports from a meta-synthesis of 16 studies exploring breast cancer survivors’ experiences with yoga, showing that yoga can improve quality of life related outcomes [39]. This might be particularly informative given the lack of significant difference in disease specific quality of life as primary outcome of the RCT [25].

Our results emphasize the importance of considering the time elapsed since treatment. Previous qualitative studies have shown variation in cancer survivors’ views of the impact of physical activity on quality of life depending on current treatment point and stage of disease [28]. As evident in some interviews, some participants had less complains and adjusted better to residual symptoms than others. This is in line with previous studies reporting lower quality of life early after treatment, followed, in the absence of disease recurrence or progression, by gradual improvements [40,41]. This might be a possible confounding effect and also lead to an unwished ceiling effect not only for yoga studies, but also for other types of interventions. Aspects regarding the timing of the intervention were also mentioned in the interviews, with some participants favoring the intervention soon after end of treatment. Future studies should therefore consider this during randomization or include it as factor in subgroup analyses comparing the effectiveness of early vs. late interventions. Furthermore, as evident in participants’ reports, the intervention effects extended beyond cancer related symptoms, including improved muscular tonus, body posture and perception, relief of back pain. This emphasizes the need of future studies to include outcomes beyond cancer related complains in order to uncover the potentially wide spectrum of yoga induced changes.

Our analysis underlines specific factors that might influence CRC survivors’ yoga practice. On one hand we have identified a series of concerns such as stoma detachment, irregular bowel activity, incontinence or reduced mobility. Similar findings were reported in the context of other physical activities [37] and differentiate CRC survivors from breast cancer survivors which report barriers centered around time-constrains, overall hindered health condition or lack of motivation [27]. These could represent potential barriers to participation in yoga classes or intervention studies and should be addressed in a sensitive manner during the recruitment process. Given our predominantly male sample, we additionally needed to overcome barriers such the preference for other forms of physical activities or gender-related perceptions [42]. On the other hand, we uncovered that CRC survivors might be particularly motivated to participate in studies as they feel that such interventions are understudied for this type of cancer, especially in comparison to breast cancer. This is most likely limited to a subsample of CRC survivors, likely highly educated and those who were searching for evidence-based interventions. However, this does not limit the applicability of this finding to simply targeting those who are already aware of the need for further research on this topic, but should encourage efforts to raise awareness on the topic and explicitly include information regarding data scarcity in the study advertisements.

Limitations associated with this study need to be acknowledged when interpreting these findings. First, all those who participated in the interviews were RCT participants enrolled in the wait-list group. Given RCT-specific eligibility criteria our trial participants may have different characteristics compared to those from a naïve cohort which could introduce bias and limit the transferability of results. Among other factors which include the transferability of these findings it is worth mentioning the relatively small sampling frame, participants were all Caucasians/Whites, they did not have any prior experiences with yoga and we do not have information on their physical activity preferences, concomitant treatments or comorbid diseases other than those covered by the RCT exclusion criteria (having no further active oncological disease, diagnosed and pharmacologically treated psychiatric disorder or adjustment disorder).

Addressing wider communities and different modes of therapy delivery would increase the transferability of the findings. In the absence of randomization, we cannot exclude a possible self-selection bias [43], such as participants experiencing more benefits being more likely to participate in the interviews. However, not all reports were positive and participants also reported no observed changes. Most of those who attended the interviews had completed the ­intervention. This limited us to explore drop-out reasons and other potential barriers that might have led to study discontinuation. Despite these limitations, this study complemented the previous reported RCT results by uncovering effects of the intervention beyond those assessed in the study as well as possible confounding factors. Furthermore, it provides a unique perspective into CRC survivors’ expectations and experiences following the yoga intervention.

Taken together, this qualitative analysis uncovered four themes present in CRC survivors’ accounts. These captured the experiences of this cancer survivorship group including varying expectations as well as perceived benefits and disease-specific challenges. This knowledge can serve to inform future studies and guide the implementation of future interventions.

## Supplementary Material

Supplemental Material

## Data Availability

The data that support the findings of this study are available from the corresponding author, H.C, upon reasonable request.
