# Supplementary material for: Yoga intervention for colorectal cancer survivors: a qualitative study exploring participants’ expectations and experiences
Source: Ann Med. 2024 Aug 30;56(1):2397571. doi: 10.1080/07853890.2024.2397571 (PMC11413962; doi:10.1080/07853890.2024.2397571)
Supplement: Supplemental Material [file IANN_A_2397571_SM3521.docx]

**Appendix A. Semi-structured interview guide**

**Part I. Interview guide before intervention**

1. Welcome and introduction

This interview is part of the study "Hatha Yoga as an adjunctive measure in patients with colorectal carcinoma". The interview will focus on your current condition and expectations related to yoga practice. The interview is expected to last no more than 30 minutes. As already discussed, the interview will be tape-recorded, later transcribed and anonymized - i.e. all names, places and statements that could allow a connection to your person will be changed. Your statements will, of course, be treated as strictly confidential.

Do you have any questions? Then let us begin the interview.

1. Narrative prompt

You will be attending your first yoga class with us today following the interview. I would therefore like to ask you a few questions about your current state of health and your expectations regarding the yoga class. There are no right or wrong answers, I am interested in everything that is important for you.

| Yoga background and general | Possible follow-up questions |
| --- | --- |
| Can you tell me what you already know about yoga? | To what extent do you know yoga from "word-of-mouth"? |
| Have you had personal experience with yoga or similar exercises before? | If yes: How long did you do 'this', what was your experience so far? |
|  | If similar practice: Expected similarities with yoga? |
| What do you expect from today's yoga class? |  |

| Yoga motivation and fears | Possible follow-up questions |
| --- | --- |
| How would you describe your interest to participate in the yoga course? | Why did you decide to participate in the course? |
| What do you think about the possibilities of yoga to influence your well-being? | What impact do you think yoga will have on your symptoms? |
| To what extent do you have or have had concerns or fears about the yoga class? |  |
| Everyday life, handling/ attitude towards the disease | Possible follow-up questions |
| To what extent do you currently have complaints? | If yes, to what extent are you affected in your daily life? |
|  | To what extent do the symptoms limit you in your daily activities or social interactions? |
|  | Can you describe specific situations in which you are limited in your daily life? |
| How would you describe your attitude towards cancer? | What feelings do you associate with cancer? |
|  | To what extent does your cancer burden you? |
|  | How would you describe your coping with your cancer? |

| Body awareness | Possible follow-up questions |
| --- | --- |
| When you focus on your body, what do you notice in particular? | Do you see a connection between how you perceive your body and cancer surgery?  Do you see other connections between the way you live your life and how you perceive your body? |
| In general, how important is it for you to feel your body? | What do you pay the most attention to? |
| Current lifestyle | Possible follow-up questions |
| When you look at your current lifestyle, what is most important to you? | Are there values or commitments that are especially important to you in your life right now? (What ideas and concerns are central to you?) |
|  | Are there relationships that seem particularly meaningful to you? |
|  | For example, do you follow a special diet? |

| Spirituality and religion | Possible follow-up questions |
| --- | --- |
| In addition to the positive health effects, yoga is often associated with spirituality.  What is your assessment in this regard? | By this, I mean that in yoga sometimes dimensions are still assumed that go beyond the purely physical. |
| Would you describe yourself as a religious, spiritual or believing person? What does this mean to you? |  |
| How important is that to you in your life? |  |
| Current feelings | |
| In addition to the more general questions about your health and expectations, I would like to conclude by asking specifically how are you feeling right now before the yoga class? | |

Thank you very much for your willingness to participate in this interview. Would you like to add anything else that would be important to you regarding the yoga course, which we have not gone into so far? (...) Then thank you again.

**Part II. Interview guide after intervention**

1. Welcome and introduction

This is the second interview in the study "Hatha Yoga as an adjunctive intervention in patients with colorectal cancer". The interview will focus on your experience of the yoga course and how you are currently feeling. The interview is expected to last no more than 45 minutes. As in the first interview, the conversation will be tape-recorded, later transcribed and anonymized, that means all names, places and statements that could allow a connection to your person will be changed. Your statements will, of course, be treated as strictly confidential.

Do you have any questions? Then let us get started with the interview.

1. Narrative prompt

Following today’s interview, you will be attending your last yoga class with us. I would therefore like to ask you about how you have experienced the yoga class over the last few weeks, as well as how you are currently feeling. There are no right or wrong answers, I am interested in everything that is important to you.

| Expectations / Motivation (Connection to the first interview) | Possible follow-up questions |
| --- | --- |
| At the beginning, we had already asked you about your expectations regarding the yoga course. Could you tell me again very briefly, what you expected from the yoga course and whether these expectations have been fulfilled? | In what ways was the course different from what you imagined? |

| Experiencing the yoga class | Possible follow-up questions |
| --- | --- |
| How did you feel about the yoga class in general? | What did you find good? (What did you find particularly good) |
|  | What didn’t you like so much? (Did something bother you?) |
|  | Can you describe how you felt in the yoga class? |
|  | To what extent did you notice differences before and after the yoga course? |
| When you think about the individual elements of the yoga class: which ones appealed to you the most and  which ones were difficult for you? | What role did the physical exercises play for you? |
|  | To what extent were some of the physical exercises difficult for you? |
|  | How did you feel about the sun salutation? |
|  | How did you perceive the breathing exercises? |
|  | How did you feel about singing the OM? |
|  | How did you experience the meditation at the end? |
|  | Which meaning had the meditation for you? |
| What was it like for you to practice yoga in a group with other patients? | How did you experience the dynamics in the group? |
|  | To what extent did the group influence how you practiced yoga? |
| How often did you practice alone at home?  What was your experience like? | To what extent do you think the frequency of practice was important? |
| Can you describe what motivated you to attend yoga classes regularly? |  |

| Symptoms/everyday life/attitudes/comparison | Possible follow-up questions |
| --- | --- |
| How do you assess the possibilities to influence your well-being through yoga today? | To what extent do you currently have any complaints? |
|  | What impact do you think yoga has had on your symptoms? |
| To what extent has your everyday life changed since you started the yoga course? | Before starting the yoga class, to what extent did your symptoms limited your daily activities or social interactions? |
|  | How has yoga practice affected these limitations? |
| Has your attitude about cancer changed since you started yoga classes? | To what extent have your feelings related to cancer changed since you started the yoga class? |
|  | To what extent does your cancer burden you today? |
|  | Did the yoga class have an impact on how you dealt with cancer? |

| Body awareness comparison | Possible follow-up questions |
| --- | --- |
| Has your body awareness changed since you started yoga classes? | What differences in your body awareness have you noticed since starting yoga classes? |
|  | How exactly would you describe these changes? |
|  | When did you first notice these changes? |
| To what extent can you make a connection between yoga practice and your body awareness? | What aspects of yoga have an impact on your body awareness? |
|  | Do you see other connections between your lifestyle and how you perceive your body? |
| How important is it for you to feel your body today? | What do you pay attention to the most? |

| Lifestyle (social environment) | Possible follow-up questions |
| --- | --- |
| When you look at your current lifestyle, what is most important to you? | Did the yoga course have an impact on how you live your life? |
|  | How has your private environment reacted to the fact that you practice yoga? Did you feel supported? |
|  | Have you noticed any changes regarding your diet? |

| Spirituality and religion | Possible follow-up questions |
| --- | --- |
| Did you perceive dimensions in the yoga class,  that went beyond the purely physical? | (Can you describe this in more detail?) |
|  | (How would you describe 'this area'?) |
|  | (What does this mean to you?) |
|  | In your opinion, are there differences between yoga and sports? |
| In the first interview we had already talked about the fact that yoga is often associated with spirituality in addition to the positive health effects, have you had any experience in this regard? | Were these experiences new to you, or do you already know them from other contexts? |
|  | Did these experiences have an impact on your everyday life? |
| Was there a connection for you between yoga and your faith? |  |
| When or where do you feel most in tune with the cosmos, or part of something bigger? |  |
| How important is 'this area' (faith, religion, spirituality) we just talked about for your life? |  |

| Concluding questions, current feelings |
| --- |
| (Can you tell me again what you think is the importance of mantra chanting and meditation in yoga)? |
| (Finally, can you tell me briefly what you think about yoga now?) |
| In addition to the questions about yoga practice and your health, I would like to conclude by asking specifically how are you feeling right now before your last yoga class? |

Thank you very much for your willingness to participate in this interview. Would you like to add anything else that would be important to you regarding the yoga course, which we have not gone into so far? (...) Then thank you again.
